# MIEN1, a novel interactor of Annexin A2, promotes tumor cell migration by enhancing AnxA2 cell surface expression

**DOI:** 10.1186/s12943-015-0428-8

**Published:** 2015-08-15

**Authors:** Marilyne Kpetemey, Subhamoy Dasgupta, Smrithi Rajendiran, Susobhan Das, Lee D. Gibbs, Praveenkumar Shetty, Zygmunt Gryczynski, Jamboor K. Vishwanatha

**Affiliations:** Department of Molecular and Medical Genetics and Institute for Cancer Research, University of North Texas Health Science Center, 3500 Camp Bowie Blvd., Fort Worth, TX 76107 USA; Institute for Cancer Research, University of North Texas Health Science Center, Fort Worth, TX 76107 USA; Texas Center for Health Disparities, University of North Texas Health Science Center, Fort Worth, TX 76107 USA

**Keywords:** MIEN1, Annexin A2, ITAM, CAAX, Migration, Invasion, Breast cancer

## Abstract

**Background:**

Migration and invasion enhancer 1 (MIEN1) is a novel gene found to be abundantly expressed in breast tumor tissues and functions as a critical regulator of tumor cell migration and invasion to promote systemic metastases. Previous studies have identified post-translational modifications by isoprenylation at the C-terminal tail of MIEN1 to favor its translocation to the inner leaflet of plasma membrane and its function as a membrane-bound adapter molecule. However, the exact molecular events at the membrane interface activating the MIEN1-driven tumor cell motility are vaguely understood.

**Methods:**

MIEN1 was first studied using in-silico analysis on available RNA sequencing data of human breast tissues and its expression was ascertained in breast cells. We performed several assays including co-immunoprecipitation, wound healing, western blotting and immunofluorescence to decipher the molecular events involved in MIEN1-mediated tumor cell migration.

**Results:**

Clinically, MIEN1 is predominantly overexpressed in Her-2 and luminal B subtypes of breast tumors, and its increased expression correlates with poor disease free survival. Molecular studies identified a phosphorylation-dependent activation signal in the immunoreceptor tyrosine based activation motif (ITAM) of MIEN1 and the phosphorylation-deficient MIEN1-mutants (Y39F/50 F) to regulate filopodia generation, migration and invasion. We found that ITAM-phosphorylation of MIEN1 is significantly impaired in isoprenylation-deficient MIEN1 mutants indicating that prenylation of MIEN1 and membrane association is required for cross-phosphorylation of tyrosine residues. Furthermore, we identified MIEN1 as a novel interactor of Annexin A2 (AnxA2), a Ca^2+^ -dependent phospholipid binding protein, which serves as an extracellular proteolytic center regulating plasmin generation. Fluorescence resonance energy transfer (FRET) confirmed that MIEN1 physically interacts with AnxA2 and functional studies revealed that they mutually cooperate to accentuate tumor cell motility. Interestingly, our study identified that ectopic overexpression of MIEN1 significantly enhances Tyr23-phosphorylation on AnxA2, thereby stimulating cell surface translocation of AnxA2 and catalyzing the activation of its proteolytic activity.

**Conclusion:**

Our data show that the presence and interaction of both MIEN1 and AnxA2 in breast tumors are crucial drivers of cell motility. Our study has now deciphered a novel regulatory network governing the vicious process of breast tumor cell invasion-metastasis, and findings suggest MIEN1-AnxA2 as prospective targets to counter the deadly disease.

**Electronic supplementary material:**

The online version of this article (doi:10.1186/s12943-015-0428-8) contains supplementary material, which is available to authorized users.

## Introduction

Migration and invasion enhancer 1(MIEN1) (also known as C35, C17orf37, RDX12, and MGC14832) is located in the chromosomal region 17q12-21, in the ERBB2 amplicon [1–4]. MIEN1 is frequently amplified along the neighboring genes, ErBB2 and GRB7 in variety of tumors including breast cancer. Our previous studies identified MIEN1 as the prime regulator of cancer cell migration and invasion [5]. In addition, we demonstrated that MIEN1 has a functional isoprenylation ‘CAAX’ motif at the C-terminal tail that is post-translationally modified by geranyl-geranyl transferase-I (GGTase-I) [6]. Prenylated MIEN1 then translocates to the inner leaflet of the plasma membrane and potentiates filopodia formation whereas prenylation-deficient MIEN1-mutants fail to migrate, invade and display reduced metastatic capacity in cancer mouse models. However, the exact molecular events at the membrane interface in MIEN1-driven breast tumor cell motility are poorly understood.

The onset of metastasis depends primarily on the ability of tumor cells to detach from basement membranes by cleaving extracellular matrix proteins and promoting motility and invasion to propel forward [7–11]. One of the key factors regulating the extracellular proteolytic process is the plasmin-plasminogen system; which is composed of a proteolytic cascade comprising the two plasminogen activators- tissue plasminogen activator (tPA) and urokinase plasminogen activator (uPA) [12–18]. Activation of this proteolytic cascade converts the inactive trypsin-like endopeptidases into potent plasmin, which then cleaves the components of the extracellular matrix proteins thereby facilitating rapid migration and invasion of tumor cells to distant organs.

Here, we report that MIEN1 regulates breast cancer cell migration and invasion in a bifunctional mechanism. We show that MIEN1 has a functional immunoreceptor tyrosine based activation motif (ITAM) cross-phosphorylated at two tyrosine-residues (Y39 and Y50), which is important for triggering downstream signal transduction. In addition, we discovered MIEN1 as a novel interacting partner of Annexin A2, a member of the Annexin family of Ca^2+^-dependent phospholipid binding proteins [19, 20]. Functional studies confirmed interaction of MIEN1 with AnxA2 at the membrane interface is necessary for activation of plasmin-plasminogen complex, thereby facilitating breast cancer cell migration and invasion. Our study identified a novel regulatory pathway for activating extracellular plasmin generation to promote enhanced breast cancer cell migration and invasion.

## Results

### MIEN1 is expressed in all subtypes of breast cancer

**Fig. 1:**
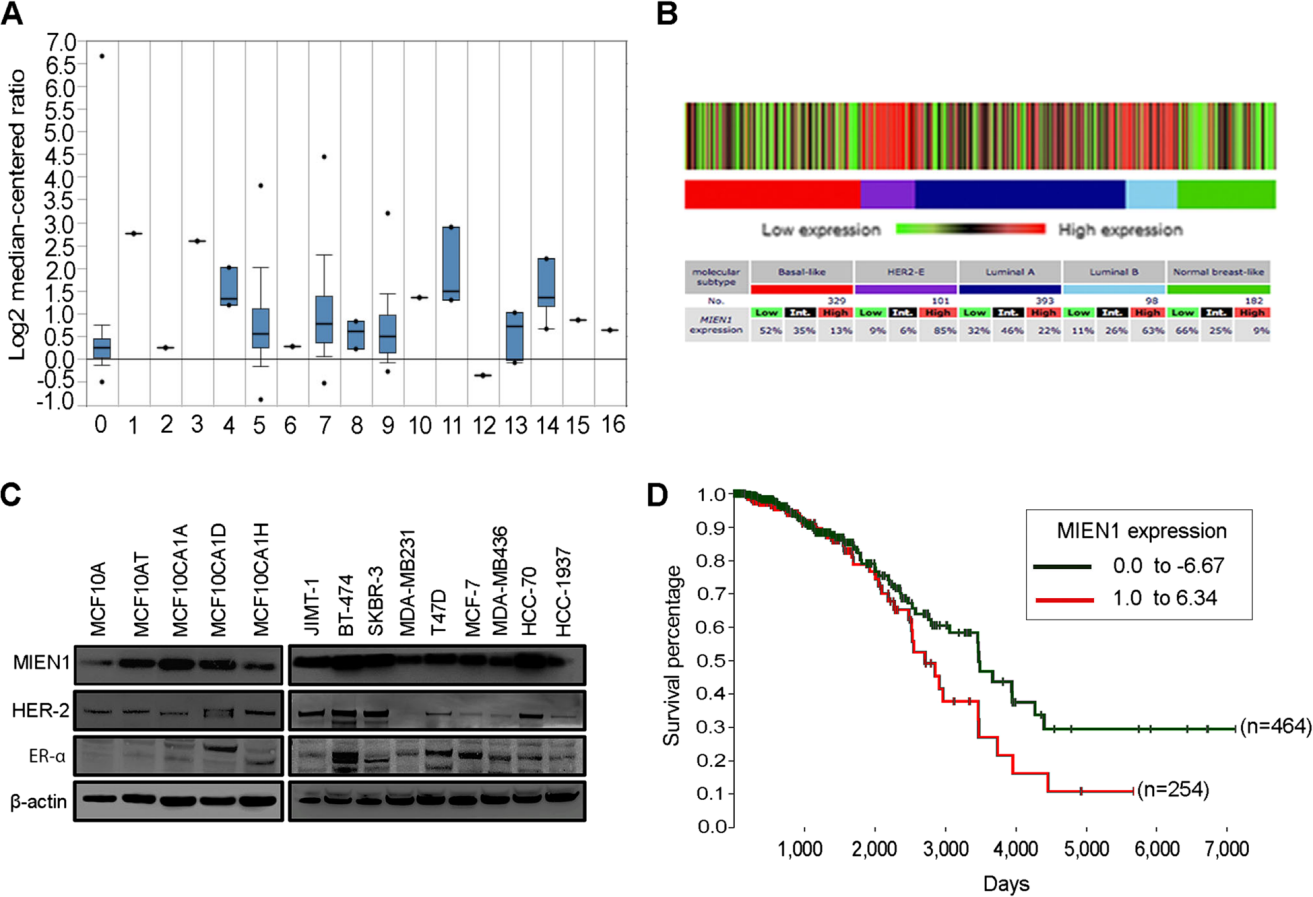
Expression of MIEN1 in breast cancer patient specimens and cultured cell lines. **a** Expression of MIEN1 in different subtypes of breast cancer patients as obtained by analyzing TCGA dataset from Oncomine. Legends and number of tissues analyzed are in parentheses- 0. No value (normal) (*n* = 61); 1. Apocrine Breast Carcinoma (*n* = 1); 2. Breast Large Cell Neuroendocrine Carcinoma (*n* = 1); 3. Ductal Breast Carcinoma (*n* = 1); 4. Intraductal Cribriform Breast Adenocarcinoma (*n* = 3); 5. Invasive Breast Carcinoma (*n* = 76); 6. Invasive Cribriform Breast Carcinoma (*n* = 1); 7. Invasive Ductal Breast Carcinoma (*n* = 392); 8. Invasive Ductal and Lobular Carcinoma (*n* = 3); 9. Invasive Lobular Breast Carcinoma (*n* = 36); 10. Invasive Papillary Breast Carcinoma (*n* = 1); 11. Male Breast Carcinoma (*n* = 3); 12. Metaplastic Breast Carcinoma (*n* = 1); 13. Mixed Lobular and Ductal Breast Carcinoma (*n* = 7); 14. Mucinous Breast Carcinoma (*n* = 4); 15. Papillary Breast Carcinoma (*n* = 1); 16. Pleomorphic Breast Carcinoma (*n* = 1). **b** MIEN1 mRNA expression in different molecular subtypes of breast cancer as determined by RSSPC using bc-GenExMiner database v3.0. A. The figure and table show the patients with low, intermediate and high MIEN1 expression in each molecular subtype. **c** MIEN1 protein expression was analyzed by immunoblotting analysis and β-actin was used as loading control. **d** Kaplan Meier survival curve showing the survival percentage of breast cancer patients were significantly low in MIEN1-high expressing tumors compared to low-expressing cohort. TCGA data analyzed using UCSD cancer genome browser

Enhanced expression of MIEN1 is reported in breast cancer compared to normal breast tissues [2]. Analysis of Cancer Genome Atlas (TCGA) data sets identified significantly elevated MIEN1 expression in different subtypes of breast carcinomas (Apocrine, Large Cell Neuroendocrine, Cribiform, Papillary, Ductal, Lobular, Mixed Ductal and Lobular, Mucinous) patients compared to normal tissues (Fig. 1a). In clinical oncology, evaluations of breast tumors are accompanied by an assessment of the molecular status of ER, PR and Her-2 oncogene. To understand the differential expression of MIEN1 in various subtypes of breast cancer, we examined the expression of MIEN1 within the molecular subtypes of breast cancer. Our findings revealed that MIEN1 is predominantly overexpressed in Her-2 positive (85 % cases with elevated MIEN1) and luminal B (63 % cases with elevated MIEN1) subtypes. However, MIEN1 expression in other subtypes basal-like and luminal A were moderate, whereas majority of the normal breast tissues had low MIEN1 (Fig. 1b). Screening of various established breast tumor lines identified increased expression of MIEN1 in majority of the breast tumor lines (MCF10AT, MCF10CA1a, MCF10CA1d, MCF10CA1h, JIMT-1, BT-474, SKBR-3, MDA-MB231, T47D, MCF-7, MDA-MB436 and HCC-70) compared to the immortalized normal mammary epithelial cell line, MCF10A (Fig. 1c). As expected most of the Her-2 amplified cell lines displayed increased expression of MIEN1 protein (CRL-2330, SKBR-3, and BT-474); but MIEN1 expression is not only restricted to Her-2 amplification indicating its distinct transcriptional and post-translational modifications contributing to its elevated expression in breast tumors. Classification of the patient cohort according to high and low MIEN1 expression using TCGA dataset, confirmed a poor survival in breast cancer patients with elevated MIEN1 expression as previously shown [21] (Fig. 1d). Altogether, these findings confirm that MIEN1 is a clinically important oncogene, and its increased expression contributes towards an aggressive disease with poor survival.

### MIEN1 has a functional ITAM-motif

**Fig. 2:**
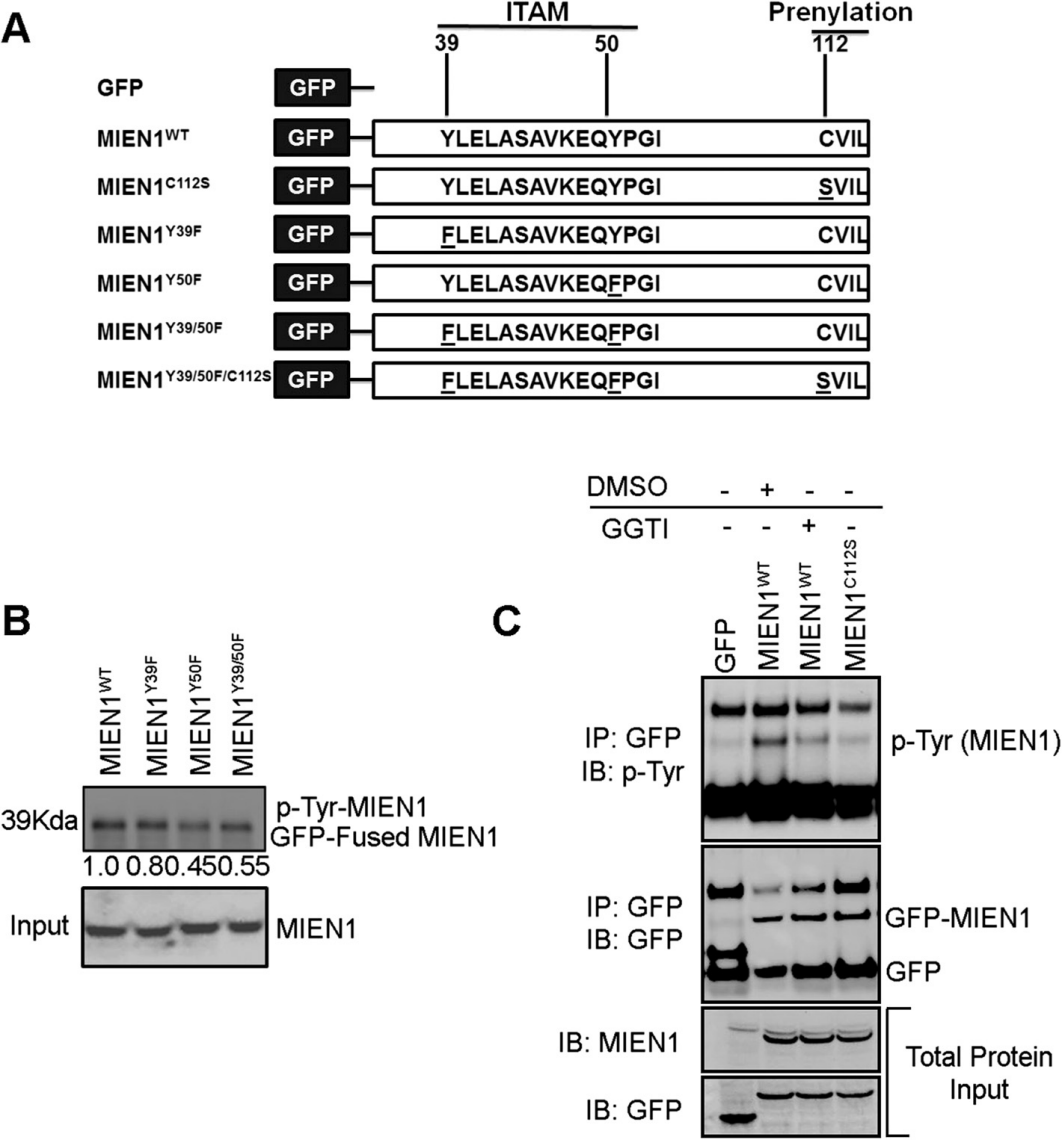
MIEN1 has a functional immunoreceptor tyrosine based activation motif. **a** Schematic representation of the ITAM and CAAX-prenylation motif on MIEN1 protein and different site directed mutants used in the current study. Tyrosine residues in ITAM domain were mutated to phenylalanine (F) and cysteine residue of the CAAX-prenylation site was mutated to serine (S). **b** NIH3T3 cells were transfected with GFP fused MIEN1^WT^, MIEN1^Y39F^, MIEN1^Y50F^ and MIEN1^Y39/50F^ constructs followed by immunoprecipitation using GFP antibody. The tyrosine phosphorylation of MIEN1 was detected using a generic phospho-tyrosine antibody. **c** NIH3T3 cells were transfected with empty GFP, MIEN1^WT^ and MIEN1^C112S^ constructs. MIEN1^WT^ transfected cells were either treated with DMSO (vehicle control) or geranylgeranyl transferase I (GGTI) inhibitor and then immunoprecipitated using GFP antibody followed by immunoblotting with phospho-tyrosine antibody. The total MIEN1 protein used for immunoprecipitation was indicated as input

In addition to isoprenylation motif [6], MIEN1 harbors several potential phosphorylation sites. Analysis of MIEN1 sequence using computational algorithms (KinasePhos 2.0) identified four potential phosphorylated tyrosine residues (Tyr29, Tyr39, Tyr50 and Tyr85) [22–25] of which Tyr39 and Tyr50 residues are located in the ITAM domain [21]. The canonical ITAM (immunoreceptor tyrosine based activation motif) is an 18 sequence amino acids (YxxI(6–8)YxxL) where tyrosine is separated from a leucine or isoleucine by any two other amino acids, giving the signature YxxL/I; and these two signatures are typically separated by 6 to 8 amino acids. To demonstrate that the Y39/Y50 in the ITAM domain of MIEN1 is phosphorylated, we investigated the phosphorylation status of MIEN1 (Fig. 2a). Immunoprecipitation of GFP-tagged MIEN1 constructs followed by immunoblot analysis using a generic phospho-tyrosine antibody, demonstrated that MIEN1 wild type (MIEN1^WT^) is tyrosine phosphorylated, whereas the Y39F and Y50F phospho-deficient mutants showed lower phosphorylation status. However Y50F mutant showed a greater loss of phosphorylation compared to Y39F (Fig. 2b). Replacement of Y39 and Y50 with phenylalanine (Y39/Y50F) reduced MIEN1-tyrosine phosphorylation by half; indicating that there is some background phosphorylation on the tyrosine residues outside the ITAM domain.

Since, MIEN1 is also modified by isoprenylation at the C-terminal ‘CVIL’ end responsible for its membrane localization [6], we investigated whether phosphorylation of ITAM-tyrosine residues is dependent on isoprenylation. For this, we analyzed the tyrosine-phosphorylation status of MIEN1 in NIH3T3 cells (with low endogenous MIEN1) transfected with GFP vector only, MIEN1^WT^ or prenylation-deficient MIEN1^C112S^ constructs. In addition, the MIEN1^WT^ expressing cells were either treated with a pharmacological inhibitor against GGTase enzyme GGTI (geranylgeranyl transferase I inhibitor) or vehicle control (DMSO). MIEN1^WT^ expressing cells showed robust tyrosine-phosphorylation whereas GGTI treatment severely abrogated the effect similar to the MIEN1^C112S^ expressing cells (Fig. 2c). These data indicate that tyrosine-phosphorylation of MIEN1 in the ITAM-domain is dependent on prior isoprenylation. These results demonstrate that correct localization of the protein to the plasma membrane is required for subsequent post-translational modification via tyrosine-phosphorylation at the ITAM-domain.

### Relative importance of ITAM and CAAX motifs in MIEN1- induced migration

**Fig. 3:**
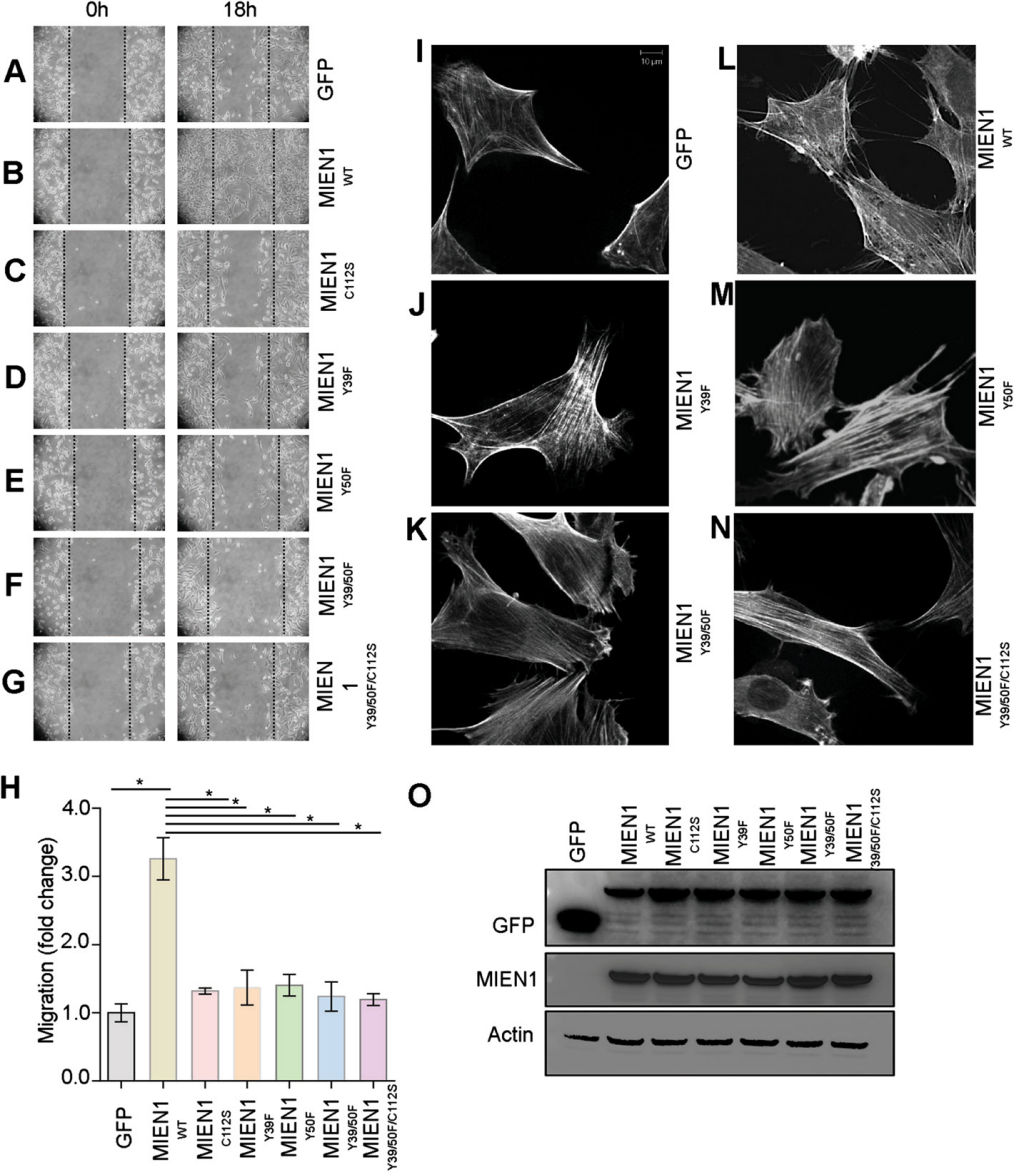
Posttranslational modifications on MIEN1 regulate its function. NIH3T3 cells transfected with the MIEN1 constructs indicated in Fig. 2a, were subjected to scratch wound assays. **a–g** Confluent monolayers of cells were wounded, and healing of the wound by cell migration was monitored for 18 h. Images were taken at 0 and 18 h. **h** The fold change in migration was normalized to GFP (empty vector) transfected cells and expressed as the means ± s.e.m of three independent experiments. **i–n** NIH3T3 cells transfected with indicated GFP-tagged MIEN1 constructs were stained with rhodamine-conjugated phalloidin post wound induction to evaluate effects of ITAM and CAAX motif on filopodia formation. **o** NIH3T3 cells expressing the GFP vector control and GFP-MIEN1 variants were analyzed for GFP and MIEN1 contents. GAPDH served as a loading control

Next, we evaluated the functional impact of MIEN1-ITAM and isoprenyl mutants compared to the wild-type protein (MIEN1^WT^). For this, we stably expressed MIEN1 constructs in NIH3T3-cells with low endogenous protein. Cell migration was significantly enhanced in cells expressing MIEN1^WT^ relative to the vector control (Fig. 3a, b), whereas MIEN1^C112S^ failed to migrate into the wound (Fig. 3c), as reported earlier [6]. Similar to the isoprenyl-mutants, NIH3T3 expressing ITAM mutants had a reduced migratory potential (Fig. 3d–f). However, we did not observe significant differences in functional contribution between individual tyrosine mutants (MIEN1^Y39F^ and MIEN1^Y50F^) compared to MIEN1^Y39/50F^ confirming that the phosphorylation of both tyrosine residues is vital for ITAM induced functions or signaling events to mediate cell migration. The MIEN1^Y39/50F/C112S^ cells did not show any additive effect suggesting both isoprenylation and ITAM-phosphorylation are important for MIEN1 function (Fig. 3g). To determine whether MIEN1 posttranslational modifications are also required for invasive function, we performed matrigel invasive assays with MDA-MB231 cells transfected with MIEN1 constructs. Consistent with the data from the migration assays, MIEN1^WT^ expression increased the invasive potential of breast cancer cells whereas ITAM and isoprenyl mutants failed to show an invasive phenotype (Additional file 1: Figure S1).

Cell migration is the result of coordination between membrane protrusive structures at a leading edge, adhesion and de-adhesion followed by translocation of the cell body in the direction of migration. In migrating cells, protrusive structures such as filopodia are the pioneers, which probe the environment for cues and guide cell migration. MIEN1 has been shown to induce filopodia formation and mutation of the isoprenyl motif impaired this function [6]. Here we inquired whether the ITAM regulates MIEN1-induced filopodia, aside from the isoprenyl motif. NIH3T3 cells expressing various constructs of MIEN1 were stained with rhodamine-conjugated phalloidin and subjected to immunofluorescence analysis post wound induction. Stable expression of MIEN1 in NIH3T3 cells visibly increased the number and length of filopodia post wound induction compared to MIEN1-mutants expressing cells (Fig. 3i–n) confirming that ITAM and CAAX are important for MIEN1 function. Immunoblotting analysis of cells expressing vector control and MIEN1 constructs confirmed the equal expression of the wild type and mutants MIEN1 protein (Fig. 3o).

### MIEN1 is an interactor of AnxA2

**Fig. 4:**
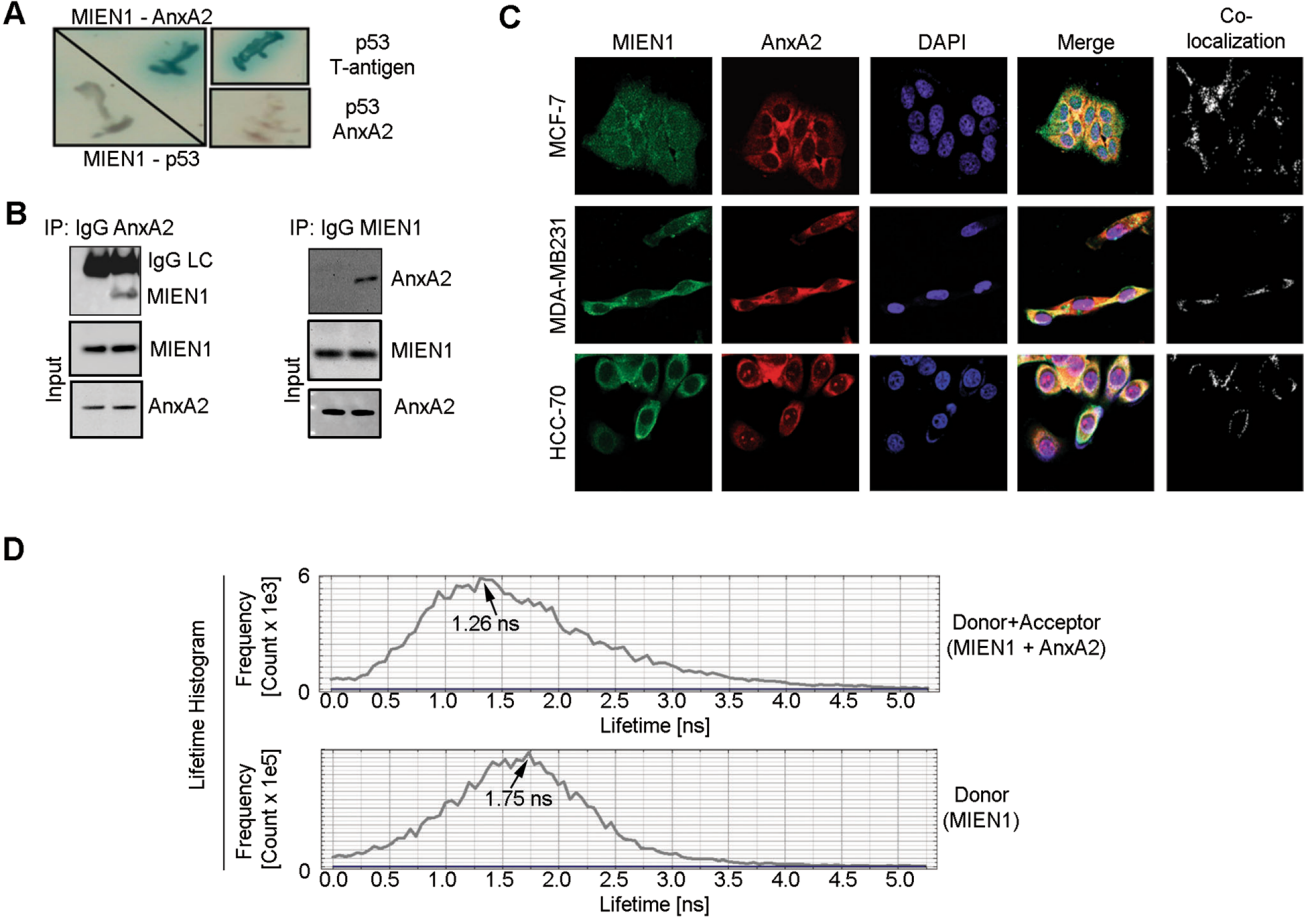
MIEN1 is a novel interactor of AnxA2. **a** MIEN1 interacts with AnxA2 in a yeast two-hybrid screen. Positive clones were selected on high-stringency medium (synthetic dropout medium) selection markers SD/−Ade/−His/−Leu/−Trp/X-α-gal, and only true interactors can activate the expression of β-galactosidase (blue color). The left panel shows positive interaction of MIEN1 with AnxA2, but not with p53. The right panel shows the positive control p53 − T-antigen interaction, while the AnxA2 − p53 interaction served as the negative control. **b** Co-immunoprecipitation of AnxA2 and MIEN1 in BT-474 cells showing the interaction of the two proteins. IgG was used as isotype control antibody and the total MIEN1 and AnxA2 used for the experiment was shown by western blotting. **c** Confocal microscopy showing co-localization of AnxA2 and MIEN1 in cancer cells. The interaction is predominantly observed around the membrane and cytosol excluding the nucleus. HCC-70, MCF-7 images were acquired with 40x objective with 1x zoom; MDA-MB231 images were acquired with 40x objective with 2x zoom. **d** FRET confirms the interaction of MIEN1 and AnxA2. Lifetime decays of the donor and donor − acceptor pair was measured to be 1.75 and 1.26 ns, respectively, and the lifetime decay histograms of the donor − acceptor pair (*upper*) and donor (*lower*) are shown

We investigated the potential interacting partners of MIEN1 to define the mechanisms associated with tumor cell migration and invasion. In a yeast two-hybrid assay, we identified MIEN1 as potential interactor of AnxA2, a Ca(2+)-dependent phospholipid binding protein which translocates to the cell surface upon cellular signaling. Full-length AnxA2 cDNA cloned into GAL4 DNA-binding domain (GAL4 DNA-BD) of vector pGBKT7 was found to interact with MIEN1 in a yeast two-hybrid screen from a transformed human placental cDNA library as bait. Positive clones were selected on high-stringency medium (synthetic dropout medium) selection markers SD/−Ade/−His/−Leu/−Trp/X-α-gal, and only true interactor-MIEN1 could activate the expression of β-galactosidase (blue color). The left panel shows positive interaction of MIEN1 with AnxA2, but not with p53. The right panel shows the positive control p53 − T-antigen interaction, while the AnxA2 − p53 interaction served as the negative control (Fig. 4a). Following immunoblotting and real-time PCR analysis of breast cell lines expressing both MIEN1 and AnxA2 (Additional file 2: Figure S2A-B), we performed co-immunoprecipitation of endogenous AnxA2 with MIEN1 in BT-474 cells to confirm the yeast two-hybrid data. The reciprocal immunoprecipitation of MIEN1 also pulled down endogenous AnxA2, confirming that these two proteins indeed reside in a complex. The total input used for the immunoprecipitation confirmed equal loading and similar levels of expression of both the proteins (Fig. 4b). Colocalization experiments using confocal microscopy also confirmed interaction of endogenous AnxA2 and MIEN1 primarily in the cytosol, plasma membrane and the perinuclear area of breast cancer cells (Fig. 4c). Finally to confirm that AnxA2 and MIEN1 physically interact intracellularly, we performed FRET detection by fluorescence lifetime imaging microscopy (FLIM) assay to measure the proximity of MIEN1 and AnxA2. The lifetime decays of the donor (MIEN1) and donor-acceptor (MIEN1-AnxA2) pair were measured to be 1.75 and 1.26 ns, respectively. Substituting the lifetime values in the Förster equation, the efficiency of energy transfer was determined to be 28 %, which corresponds to a distance of 50.3 Å between the donor and acceptor pair; indicating that MIEN1 and AnxA2 indeed physically interact and reside in a very close proximity (Fig. 4d). These studies clearly validate MIEN1 as a novel interactor of AnxA2.

### MIEN1 enhances AnxA2 phosphorylation to promote cell surface translocation and breast cancer cell migration

**Fig. 5:**
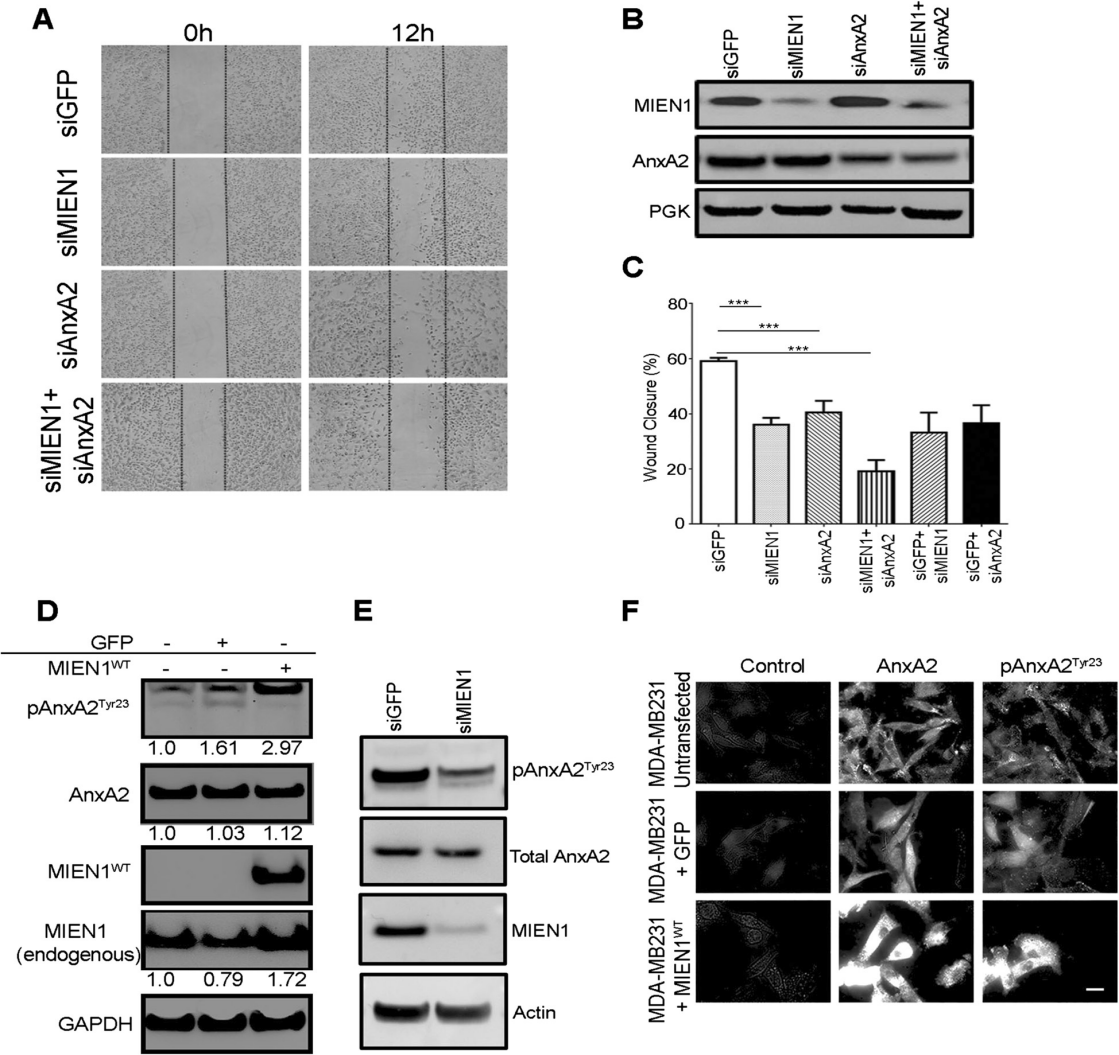
MIEN1 activates translocation of AnxA2 to plasma membrane to promote breast cancer cell migration. **a** Control and MIEN1 transfected MDA-MB231 cells were subjected to western blot analysis with anti-MIEN1 or anti-AnxA2 antibody. PGK served as a loading control. **b** Down-regulation of MIEN1 and AnxA2 reduces the abilities of MDA-MB231 breast cancer cells to migrate in vitro. Confluent MDA-MB231 monolayers transfected with control GFP, AnxA2 or/and MIEN1 were wounded using a pipet tip, 60 h after transfection. Following the wound formation, plates were incubated for 12 h and the wound closure areas were visualized on an Olympus Microscope (Carl Zeiss). Representative images were acquired at 0 and 12 h. **c** Quantification of cell migration was achieved using Image J software. Data are presented as percentages of the recovered scratch area relative to untreated control cells. Columns are the means five replicates from two independent experiments and bars are s.d. **d** MDA-MB231 and MDA-MB231 cells stably transfected with control GFP or GFP-MIEN1^WT^ were subjected to western blot analysis with anti-phosho AnxA2, anti-AnxA2 and anti-MIEN1 antibodies. GAPDH served as a loading control. The quantification of the representative blots is the densitometric average of three independent experiments analyzed using ImageJ and normalized to GAPDH. **e** Control and MIEN1 transfected MDA-MB231 cells were subjected to western blot analysis with anti-phosho AnxA2, AnxA2, anti-MIEN1 and actin as loading control. **f** Representative images, as captured using a 60X oil immersion TIRF microscope, of MDA-MB231 and MDA-MB231 cells stably transfected with GFP-MIEN1^WT^ grown to sub-confluence on coverslips, fixed with PFA, unpermeabilized, and processed for TIRF microscopy with the specific antibodies. At least three independent fields were analyzed for confirmation of the staining pattern represented. Scale bar denotes 20 μm

We investigated the effects of MIEN1 and AnxA2 interaction on cell migration, given their independent implications in cell migration and invasion (Fig. 5a-c). We silenced MIEN1 and AnxA2 alone or in combination (Fig. 5b) and examined the effects on motility of MDA-MB231 cells. In monolayer migration assays, the wound closure of cells with MIEN1 knockdown was significantly inhibited compared to siGFP (control) transfected MDA-MB231 cells (Fig. 5a, Additional file 3: Figure S3A-B). Silencing of AnxA2 resulted in impairment of cell motility at 6 and 12 h following wound creation (Fig. 5a, Additional file 3: Figure S3A-B). Using similar knockdown strategy, we observed that dual depletion of MIEN1 and AnxA2 led to a two-fold decrease compared to siGFP (Fig. 5c), indicating that MIEN1 and AnxA2 functionally cooperate to promote breast cancer cell migration. AnxA2 is a phospholipid binding protein localizing to the plasma membrane towards the cytosolic side. Previous studies proved that the phosphorylation of AnxA2 at the Y23 residue in the N-terminus of the protein causes its translocation to the extracellular surface, thereby activating extracellular proteolysis [26, 27]. To verify whether MIEN1 expression affects AnxA2 phosphorylation and subsequent translocation to the extracellular surface, we performed immunoblotting analysis followed by total internal reflection microscopy (TIRF-M). As shown in Fig. 5d, over-expression of MIEN1 enhanced AnxA2 phosphorylation at Y23. Conversely, down-regulation of MIEN1 led to a decreased phosphorylation of AnxA2 at Y23 (Fig. 5e). To further confirm this observation, we performed total internal reflection fluorescence microscopy (TIRF-M) in MDA-MB231 cells. Stable MDA-MB231 cells expressing vector control or MIEN1^WT^ were probed with AnxA2 and phospho-AnxA2^Tyr23^ antibodies followed by TIRF microscopy to determine cell surface total-AnxA2 and phospho-AnxA2. MIEN1 overexpression significantly enhanced phosphorylation of AnxA2 at the tyrosine 23 residue and subsequently cell surface localized AnxA2 levels; indicating MIEN1 dependent signaling and interaction activate AnxA2 function (Fig. 5f).

### MIEN1 activates AnxA2 dependent extracellular proteolytic functions

**Fig. 6:**
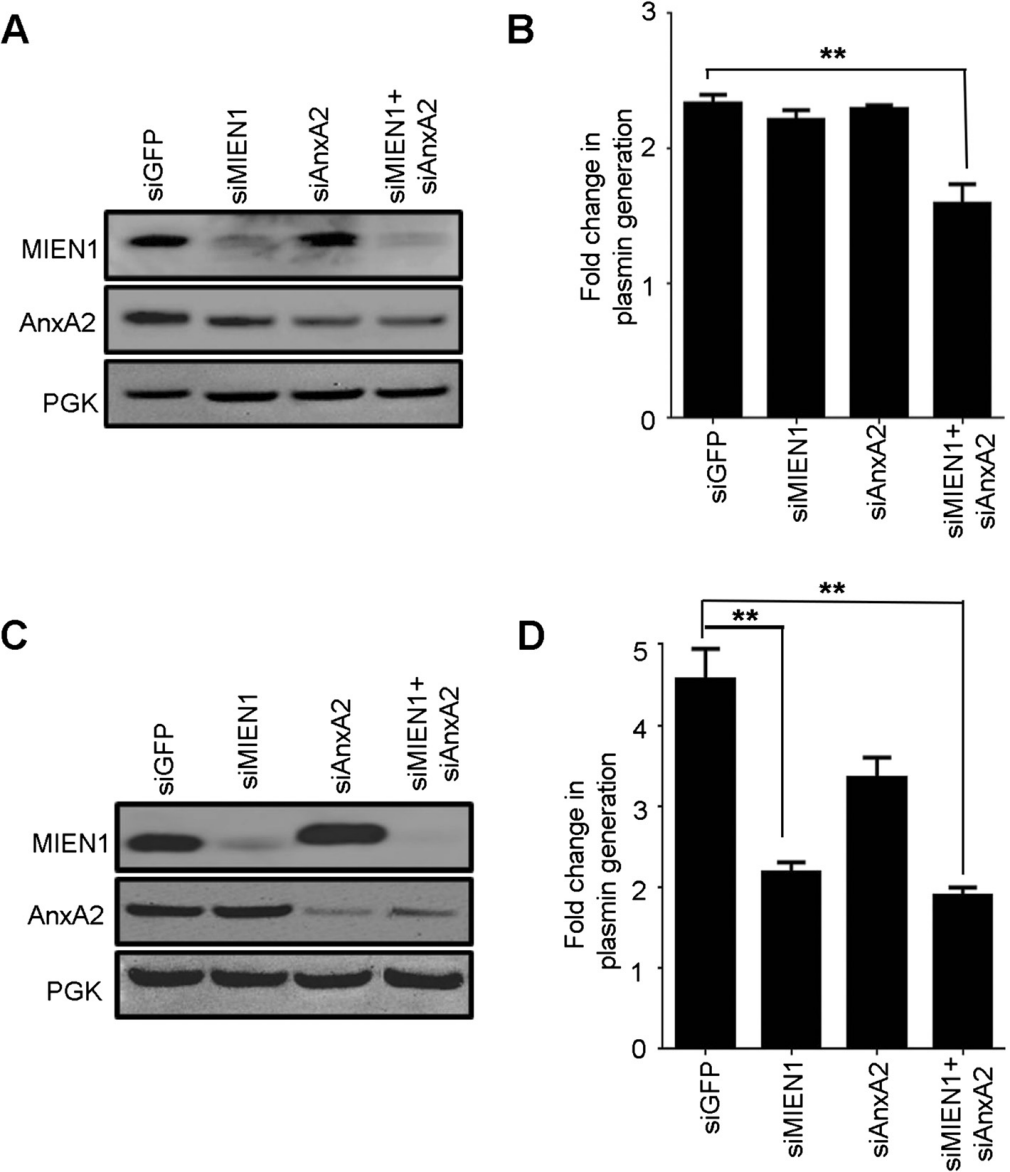
AnxA2 and MIEN1 silencing inhibit tPA dependent plasmin generation. Plasmin activity was determined at 460 nm using recombinant plasminogen, TPA and fluorogenic plasmin substrate D-VLK-AMC. Western blotting was performed to confirm depletion of MIEN1 and AnxA2 in HCC-70 (**a**) and MCF-7 (**c**) cells following siRNA knockdown. PGK served as a loading control. Total fold change in plasmin level in HCC-70 (**b**) and MCF-7 (**d**) cells was calculated by normalizing the initial rates of plasmin in untreated cells (which were assigned a value of 1). The data is presented as means ± s.d. (*n* = 6 for untreated controls and *n* = 6 for siRNA treatments)

Extracellular surface localized AnxA2 functions as a receptor for tPA and plasminogen; by binding to both enzymes, and catalytically activating plasmin generation [28, 29]. Increased levels of plasmin enhance proteolytic cleavage of ECM thus allowing cancer cells to migrate and invade to distant sites. To confirm that MIEN1 regulates AnxA2 dependent plasmin generation, we investigated the effects of MIEN1 ablation either alone or in combination with AnxA2 plasmin generation in breast cancer cells. Using basal-like HCC70 and luminal MCF7 cells (these two lines express high levels of both AnxA2 and MIEN1), we inquired whether the interaction of MIEN1 and AnxA2 had an effect on plasmin generation. We first confirmed siRNA-mediated knockdown of MIEN1 and AnxA2 in HCC-70 and MCF-7 (Fig. 6a, c) respectively. Western blotting analysis confirmed that the expression of MIEN1 or AnxA2 was dramatically reduced in the cells upon transfection with either MIEN1 or AnxA2 siRNA. Next, we examined the biochemical conversion of plasminogen to plasmin in both cell lines upon siRNA treatment. Silencing of both MIEN1 and AnxA2 led to a significant decrease in plasmin levels in both HCC-70 and MCF-7 (*P* < 0.05) (Fig. 6b, d). While the knockdown of AnxA2 led to a decrease in plasminogen conversion to plasmin in the initial hours as previously shown [30], the total change in plasmin levels were insignificant compared to the control siRNA treated cells. Interestingly, suppression of MIEN1 in MCF-7 cells led to approximately 1.7 fold decrease in plasmin levels but not in HCC-70; a difference which can be attributed to the expression of various regulators of the plasminogen-plasmin system. Taken together, these data indicate that co-expression of both AnxA2 and MIEN1 enhance plasmin generation and lead to an increase in breast tumor cell migration and invasion which in turn drive the metastatic process.

## Discussion

Cell motility process is a highly coordinated event integrated by extensive transient signaling networks of proteins [31, 32]. Protein–protein interactions are the underlying backbone of migratory processes including actin structures, proteolytic enzymes that degrade the ECM and the coordination between detachment/re-adhesion cycles [33]. Here we report the dynamics of MIEN1-induced signaling that regulate tumor cell migration and invasion.

MIEN1 expression is significantly increased in breast cancer cells and analysis of TCGA database showed that elevated expression of MIEN1 correlates with poor survival of breast cancer patients. Several post-translational modifications regulate MIEN1 protein functions and downstream signaling events. In addition to isoprenylation, in this study we identified that phosphorylation of tyrosine residues (Y39 and Y50) in the ITAM motif is important for its function. Signal transduction by ITAM is initiated by the phosphorylation of the tyrosine residues within the ITAM sequence, followed by the recruitment of members of the Syk kinase family [34–36]. The binding of Syk to the phosphorylated-ITAM results in the activation of multiple signaling cascades. Previous studies indicated that Syk kinase potentiates MIEN1 dependent signaling in breast tumor cells [21]. Using phospho-deficient single or double mutants, we show that MIEN1 is tyrosine phosphorylated in the ITAM motif. In addition, we also demonstrate that isoprenylation and membrane localization is important for ITAM-phosphorylation. Blocking isoprenylation of MIEN1 using GGTase inhibitors or genetic mutation of the ‘CVIL’ prenylation domain significantly impairs ITAM-or tyrosine phosphorylation. These findings indicate that ITAM-dependent signal transduction by MIEN1 depends on prior isoprenylation and correct localization of the protein to the inner leaflet of plasma membrane. As expected, ITAM phosphorylation was also important for MIEN1-dependent migration and invasion as phospho-deficient MIEN1 mutants severely impaired breast cancer cell migration and invasion.

Interestingly, we identified MIEN1 as an important interactor of AnxA2. A multifunctional protein, AnxA2 is involved in various cellular activities and its dysregulation is implicated in multiple diseases including breast cancer [37]. Although, AnxA2 is predominantly a cytosolic protein, phosphorylation on its N-terminal Y23 translocates the protein to the cell surface [38, 39]. Cell surface AnxA2 interacts with plasminogen and tPA and mediates the conversion of plasminogen to plasmin [40]. In breast cancer, AnxA2 mediated plasmin activation is shown to be essential for angiogenesis, migration and invasion which are critical events in disease metastasis.

Our data provide evidence that MIEN1 interaction with AnxA2 enhances Y23 phosphorylation of AnxA2 and subsequent translocation of the protein to the cell surface leads to increased plasmin levels which support breast tumor cell motility. Although, MIEN1 does not have a kinase domain, we believe this phosphorylation on AnxA2 is a downstream signaling event regulated by MIEN1.

## Conclusion

Our studies convincingly demonstrate that interaction of MIEN1 with AnxA2 is required for extracellular plasmin generation thereby increasing breast cancer cell migration and invasion. Our findings place MIEN1 and anxA2 as attractive therapeutic targets for blocking invasive cancers.

## Materials and methods

### In silico analysis

MIEN1 was first studied using in-silico analysis on available DNA microarray and RNA sequencing data of breast tumors and normal tissues from TCGA website (http://cancergenome.nih.gov/) and the bc-GenExMiner database v3.1 (http://bcgenex.centregauducheau.fr/BC-GEM/GEM_Accueil.php?js=1). The breast cancer gene-expression Miner v3.1 was utilized to assess MIEN1 expression in breast cancer molecular subtypes. Bc-GenEXMiner analyzes available gene expression data sets from repositories such as Gene Expression Omnibus (GEO), Array Express and Stanford microarray database [41–43]. The analysis was performed through a built-in robust single sample predictor (SSP) classification (RSSPC), which includes intersection of three SSPs [44]. The UCSC Cancer Genomics Browser can be accessed through https://genome-cancer.ucsc.edu/ and following the central hyperlink. The UCSC Cancer Genomics Browser displays the genomic, clinical and annotation data in multiple views [45–50]. We used the breast cancer gene expression (BRCA) (IlluminaHiSeq) dataset followed by subgrouping for C17orf37 (MIEN1) expression. Subgroups allow users to group samples according to an arbitrary combination of annotation data values. We stratified the BRCA dataset according to MIEN1 expression: low (green from 0.0 to −6.67) and high (red from 1.0 to 6.34) followed by Kaplan Meier plot to generate the survival percentage over time (days) with respect to MIEN1 expression.

### Plasmids

GFP-MIEN1 (MIEN1^WT^) was generated by PCR amplification of full-length human MIEN1 cDNA (347 base pairs) and directionally cloned into pEGFP-C1 vector as previously described [5, 6]. GFP-MIEN1 mutants were generated using QuikChange multi-site-directed mutagenesis kit from Stratagene (La Jolla, CA, USA) from the GFP-MIEN1^WT^ template.

### MIEN1 and AnxA2 small interfering RNA transfection in breast cancer cells

Human MIEN1 smart pool siRNA and AnxA2 smart pool siRNA were used for knock-down experiments (Dharmacon, Lafayette, CO). Green fluorescent protein (GFP) siRNA (Qiagen, Germatown, MD) served as control. Breast cancer cells were transfected with siRNA duplex at a concentration of 40 nM using Lipofectamine RNAiMAX purchased from Invitrogen (Grand Island, NY, USA).

### Cell lines and culture conditions

NIH3T3 mouse fibroblast cells and human breast cancer cell lines (SKBR-3, BT-474, MDA-MB231, MDA-MB4-36, HCC-70, HCC-1937, MCF-7, T47D) were obtained from American type culture collection. MCF10A, MCF10AT, MCF10CA1a, MCF10CA1d, MCF10CA1h cells were obtained from Karmanos cancer center, and JIMT-1 from Leibniz-Institut DSMZ. All cell lines were maintained in a humidified incubator containing 5 % CO_2_/95 % air at 37 °C. Stable NIH3T3 and MDA-MB231 cells expressing GFP-MIEN1 constructs were generated using 500 μg/ml and 650 μg/ml G418 antibiotic (Invitrogen).

### Yeast two-hybrid screening

A yeast two-hybrid screening was carried out as described previously. Full-length human AnxA2 cDNA (GenBank entry NM_004039) fragment was cloned into pGBKT7 vector (Clontech). The recombinant plasmid used was GAL4 DNA binding bait (DNA-BD/bait) and transformed into AH109 yeast cells screened for growth on synthetic dropout (SD/-Trp) medium. A human placental pre-transformed cDNA library (Clontech) was used for screening interacting proteins. This library was made using the recombinant vector pGADT7, which contains the activation domain (DNA-Ac/library) with a different nutrient marker -Leu and was transformed into strain Y187 and screened for growth on SD/-Leu medium. The interaction screening was conducted by mating the DNA-BD/ bait strain and DNA-Ac/library strain, and the positive clones were selected. Positive clones were selected on high-stringency medium with selection markers SD/-Ade/-His/-Leu/-Trp/ X-R-gal and then screened as described above (Clontech). Positive yeast clones were selected by prototrophy for histidine or expression of β-galactosidase and then subjected to sequence analysis to search for novel interacting proteins.

### FLIM based forster resonance energy transfer (FRET) assay

FRET was performed as described previously with minor modifications [51]. Briefly, cells were immunostained for MIEN1 and AnxA2, labeled with donor Alexa-488 and acceptor fluorophore Alexa-594, respectively. Upon transfer of energy from the donor to the acceptor, the donor fluorophore’s lifetime was monitored as an indicator for the presence or absence of FRET.

### Determination of the förster distance (R_0_)

The transfer efficiency was assessed from the donor (D) and the donor in the presence of the acceptor (D + A) lifetimes using the equation below

$$ \mathrm{E} = \frac{1\hbox{-} \left(\mathrm{D}+\mathrm{A}\right)}{\mathrm{D}} = \frac{1\hbox{-} 1.2}{1.75} = 0.28 $$

Using the transfer efficiency, E (0.28) and the transfer distance, R0 (43 A^ο^), the distance between the two molecules was determined to be 50.3A^ο^

$$ \mathrm{E} = \frac{{\left({\mathrm{R}}_0\right)}^6}{{\left({\mathrm{R}}_0\right)}^6 + {\left(\mathrm{r}\right)}^6} = \frac{0.28 = {(43)}^6}{(43)^6 + {\left(\mathrm{r}\right)}^6} = 50.3{\mathrm{A}}^{\circ } $$

### Immnunoprecipitation and immunoblotting assays

Immunoprecipitation and immunoblotting were performed as described previously [51]. In brief cells were lysed with NP-40 lysis buffer supplemented with protease and phosphatase inhibitor cocktail (Millipore, Billerica, MA). The supernatants were taken as the total cell lysates; and using protein A/G PLUS-Agarose beads (Santa Cruz Biotechnology, Santa Cruz, CA), AnxA2 and MIEN1 were immunoprecipitated. The immunoprecipitated proteins were separated by SDS-PAGE and transferred to nitrocellulose membranes for immunoblotting. The antibodies used were MIEN1 from Abnova (Taipei, Taiwan), AnxA2 (Santa Cruz Biotechnology) and β-actin (Santa Cruz Biotechnology)

### Migration and invasion assay

Cells were grown in a monolayer and upon reaching confluence, the cell layer was scratched using a sterile micropipette tip. The wound was imaged at the indicated time points following wound creation. Representative images were captured and analyzed using Image J software. Invasion assays were performed using HTS FluoroBlok multi well insert system from BD Falcon, (San Jose, CA, USA) as previously described [6].

### Immunofluorescence

Cells were fixed using 4 % paraformaldehyde (Affymetrix, Santa Clara, CA, USA) and stained with rhodamine-conjugated phalloidin, MIEN1 and AnxA2 primary antibodies followed by Alexa Fluor 488 and 594 secondary antibodies (Invitrogen). Cells were mounted on glass coverslips with Prolong Gold mounting medium (Invitrogen). Confocal images were acquired using Zeiss confocal microscope LSM510.

### Total internal reflection fluorescence (TIRF) microscopy

For TIRF microscopy, MDA-MB231 cells were grown on coverslips and then transfected with empty vector or GFP-MIEN1 wild type. The cells were fixed with 4 % paraformaldehyde, permeabilized with 0.1 % Triton X-100, washed with PBS and blocked with BSA. Cells were subsequently treated with AnxA2 (BD Biosciences) and phospho-AnxA2 Tyr23 (Santa Cruz Biotechnology) antibodies followed by AlexaFlour 488 conjugated secondary antibody and mounted with Permafluor mounting medium. For TIRF images, the cells were visualized on an Olympus IX71 microscope with commercial TIRF attachment as described previously by 60 × oil immersion objective [5].

### Plasminogen activation assays

The assay was performed according to the previously established protocols [26]. In brief, HCC-70 and MCF-7 cells were transfected with GFP, MIEN1 and/or AnxA2 siRNA. Cells were then incubated with recombinant 10 nM human tPA and 100 nM Glu-plasminogen and subsequently treated with the fluorogenic plasmin substrate, D-VLK-AMC (D-Val-Leu-Lys-7-amido-4-methylcoumarin; Sigma). Substrate hydrolysis was measured for 12 h. Results were obtained as fluorescence units (FUs) using a fluorescence spectrophotometer (380 nm excitation and 460 nm emission wavelengths).

### Statistical analyses

Data acquired from migration, invasion, immunofluorescence and plasmin generation assays were analyzed using student *t*-test. The statistical analysis was performed using Windows version of SPSS along with GraphPad Prism 4.02 software. The ImageJ software was used for wound healing assay analysis. Significance was defined as *P < 0.05, **P < 0.01, ***P < 0.001 compared to controls and between different treatment groups.

## Additional files

Additional file 1: Figure S1. Posttranslational modifications on MIEN1 regulate its invasion function. (A-G) MDA-MB231 transfected cells were prelabeled with calcein AM fluorescent dye and an invasion assay was performed with 10 % serum as a chemoattractant in the lower chamber. The fold change of invasion was normalized to GFP (empty vector) transfected cells and expressed as the means ± S.E. of three independent experiments (O). (TIFF 11758 kb) Additional file 2: Figure S2. Expression of MIEN1 and AnxA2 in a panel of breast cell lines. (A) The protein expressions of MIEN1 and AnxA2 were analyzed by immunoblotting analysis. (B) Quantitative real time PCR showed the mRNA expressions of MIEN1 and AnxA2 in breast cell lines. (JPEG 342 kb) Additional file 3: Figure S3. MIEN1 and AnxA2 are required for MDA-MB231 cell migration. MDA-MB231 cells were treated with AnxA2 or/and MIEN1 siRNA/GFP siRNA for 72 h. Scratch was made using a pipet tip in MDA-MB231 cells confluent monolayer and healing of the wound was monitored. (A) Representative images were acquired at 0 and 6 h. (B) Graph shows the percent wound closure at 6 h intervals after wound formation. (JPEG 913 kb)
