# Supplementary figures and images for: MIEN1, a novel interactor of Annexin A2, promotes tumor cell migration by enhancing AnxA2 cell surface expression
Source: Mol Cancer. 2015 Aug 15;14:156. doi: 10.1186/s12943-015-0428-8 (PMC4536591; doi:10.1186/s12943-015-0428-8)

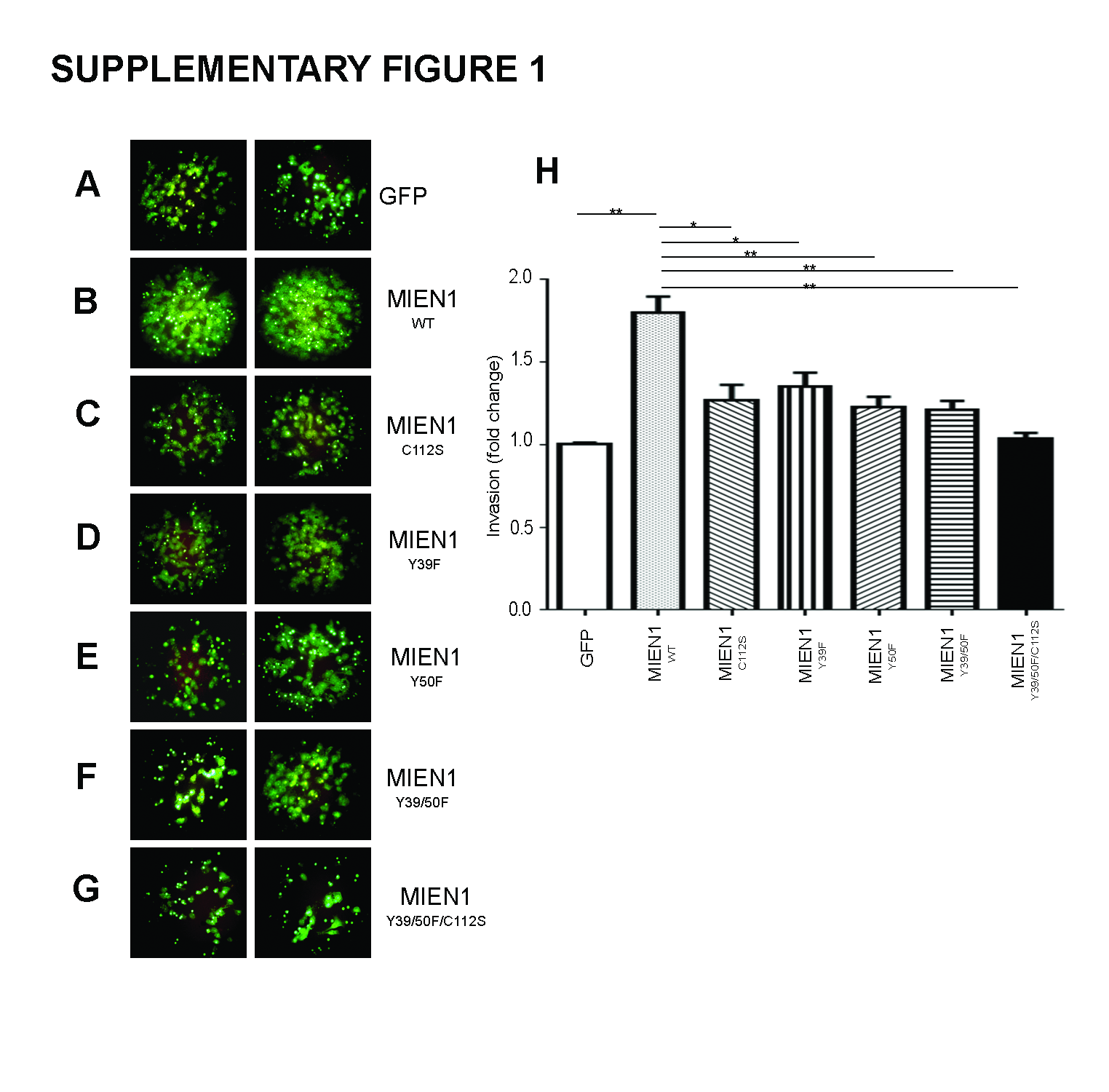

Supplement: Additional file 1: Figure S1. — Posttranslational modifications on MIEN1 regulate its invasion function. (A-G) MDA-MB231 transfected cells were prelabeled with calcein AM fluorescent dye and an invasion assay was performed with 10 % serum as a chemoattractant in the lower chamber. The fold change of invasion was normalized to GFP (empty vector) transfected cells and expressed as the means ± S.E. of three independent experiments (O). (TIFF 11758 kb) [file 12943_2015_428_MOESM1_ESM.tif]

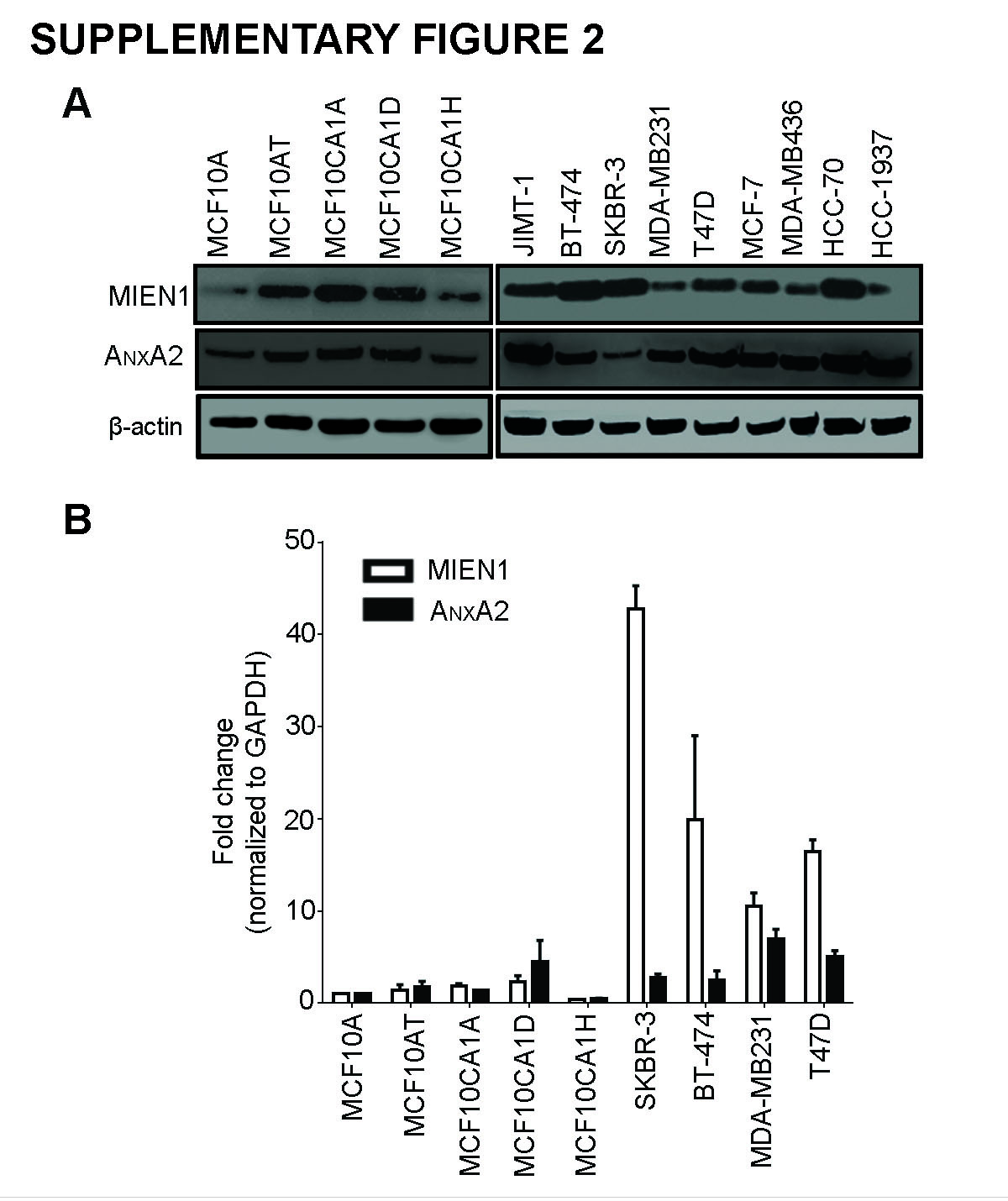

Supplement: Additional file 2: Figure S2. — Expression of MIEN1 and AnxA2 in a panel of breast cell lines. (A) The protein expressions of MIEN1 and AnxA2 were analyzed by immunoblotting analysis. (B) Quantitative real time PCR showed the mRNA expressions of MIEN1 and AnxA2 in breast cell lines. (JPEG 342 kb) [file 12943_2015_428_MOESM2_ESM.jpg]

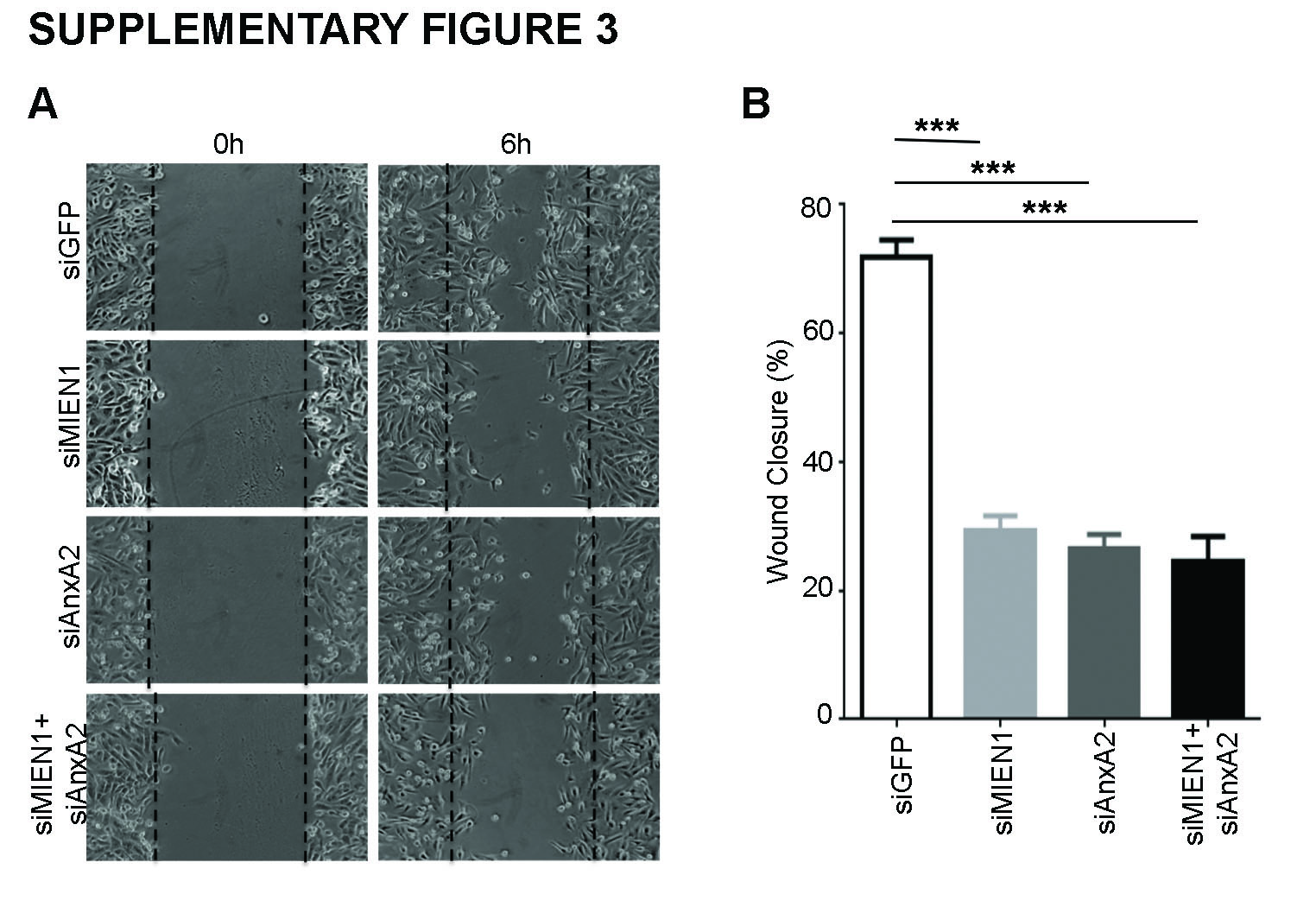

Supplement: Additional file 3: Figure S3. — MIEN1 and AnxA2 are required for MDA-MB231 cell migration. MDA-MB231 cells were treated with AnxA2 or/and MIEN1 siRNA/GFP siRNA for 72 h. Scratch was made using a pipet tip in MDA-MB231 cells confluent monolayer and healing of the wound was monitored. (A) Representative images were acquired at 0 and 6 h. (B) Graph shows the percent wound closure at 6 h intervals after wound formation. (JPEG 913 kb) [file 12943_2015_428_MOESM3_ESM.jpg]
